# Supplementary figures and images for: Hemogram data as a tool for decision-making in COVID-19 management: applications to resource scarcity scenarios
Source: PeerJ. 2020 Jun 29;8:e9482. doi: 10.7717/peerj.9482 (PMC7331623; doi:10.7717/peerj.9482)

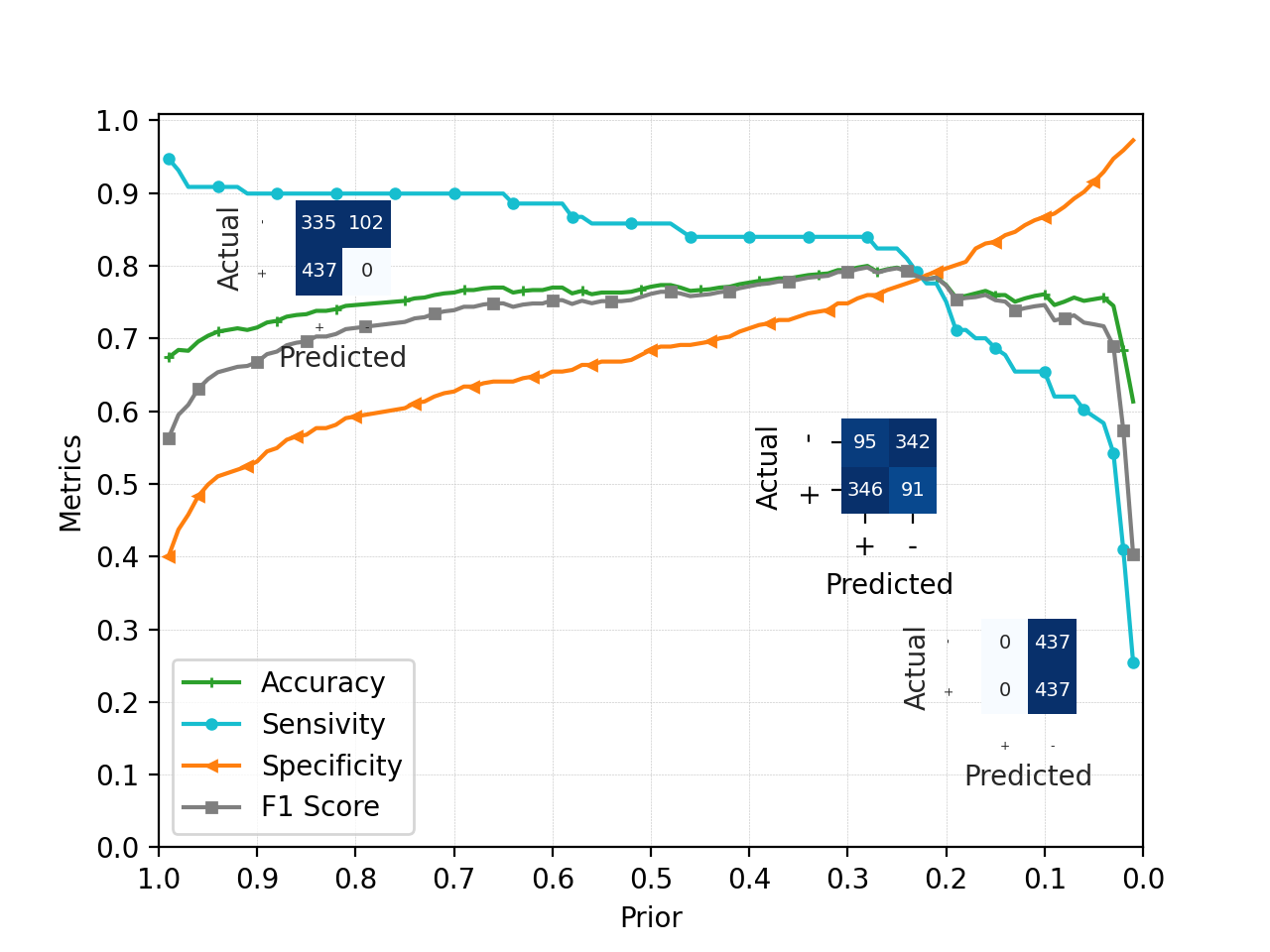

Supplement: Supplemental Information 2 — In this case, random oversampling of positive results was employed, until sample number in each class is identical. Prior probabilities are presented in reference to positive qRT-PCR prediction. Confusion matrices (left to right) are presented for 0.9999, 0.2237 and 0.0001 prior probabilities, respectively. Sensitivity=True Positive Ratio; Sensitivity=True Negative Ratio. Random seed was set to 0 for replication purposes. [file peerj-08-9482-s002.png]
